# Neuroimaging of amblyopia and binocular vision: a review

**DOI:** 10.3389/fnint.2014.00062

**Published:** 2014-08-06

**Authors:** Olivier Joly, Edit Frankó

**Affiliations:** ^1^MRC Cognition and Brain Sciences UnitCambridge, UK; ^2^Department of Experimental Psychology, University of OxfordOxford, UK; ^3^Department of Neurodegenerative Disease, Institute of Neurology, University College LondonLondon, UK; ^4^National Prion Clinic, National Hospital for Neurology and Neurosurgery, University College London HospitalsLondon, UK

**Keywords:** amblyopia, binocular vision, stereopsis, visual cortex, neuroimaging

## Abstract

Amblyopia is a cerebral visual impairment considered to derive from abnormal visual experience (e.g., strabismus, anisometropia). Amblyopia, first considered as a monocular disorder, is now often seen as a primarily binocular disorder resulting in more and more studies examining the binocular deficits in the patients. The neural mechanisms of amblyopia are not completely understood even though they have been investigated with electrophysiological recordings in animal models and more recently with neuroimaging techniques in humans. In this review, we summarize the current knowledge about the brain regions that underlie the visual deficits associated with amblyopia with a focus on binocular vision using functional magnetic resonance imaging. The first studies focused on abnormal responses in the primary and secondary visual areas whereas recent evidence shows that there are also deficits at higher levels of the visual pathways within the parieto-occipital and temporal cortices. These higher level areas are part of the cortical network involved in 3D vision from binocular cues. Therefore, reduced responses in these areas could be related to the impaired binocular vision in amblyopic patients. Promising new binocular treatments might at least partially correct the activation in these areas. Future neuroimaging experiments could help to characterize the brain response changes associated with these treatments and help devise them.

## INTRODUCTION

Amblyopia is the reduction of best-corrected visual acuity to less than 6/9 in Snellen optotype or at least two-line difference in LogMAR optotype between the eyes. This measured reduction in visual acuity cannot be directly related to structural abnormalities of the eye and cannot be corrected by spectacle glasses alone. Amblyopia is often considered as a monocular disorder. Indeed, it usually affects one eye, although rarely it can be bilateral, and it is the most common cause of monocular blindness. The prevalence of amblyopia is 2–4% in the general population (Donnelly et al., 2005; Robaei et al., 2006; Williams et al., 2008). Amblyopia is believed to be caused by abnormal visual experience during the critical period of visual development in early life (first 7–10 years). It is mainly associated with strabismus or anisometropia, more rarely with visual deprivation arising from ptosis or congenital cataract.

The three most common types of amblyopia are strabismic, anisometropic, and combined mechanism (both strabismus and anisometropia are present) amblyopia. The prevalence of these different types seems to depend on the age; in children under the age of three, amblyopia affects about 50% of the children suffering from strabismus and about 18% of the children with anisometropia (Birch and Holmes, 2010). However, this ratio seems to reverse in adults; Attebo et al. (1998) found that in 50% of the patients the cause of amblyopia was anisometropia whereas strabismus was responsible only in 19% of the cases. A possible explanation for this difference in prevalence is that anisometropia may develop later, or it may require longer duration to cause amblyopia (Birch, 2013). The different types of amblyopia are also characterized by different patterns of visual acuity and contrast sensitivity loss. Strabismic amblyopia results in moderate acuity loss and increased contrast sensitivity at low spatial frequency, whereas anisometropic amblyopia causes moderate acuity loss and decreased contrast sensitivity. In combined mechanism amblyopia the acuity is usually very poor whereas the contrast sensitivity is normal or slightly reduced (McKee et al., 2003). It was also shown that the reduction in contrast sensitivity is disproportionally higher for high as compared to low spatial frequencies (Hess et al., 1978; Bradley and Freeman, 1981; Hess and Pointer, 1985). Importantly, visual acuity in amblyopia was also found to correlate with residual binocular function; patients with no residual binocular function generally have poorer acuity (McKee et al., 2003). The defect in stereopsis also depends on the type of amblyopia; it is more often disrupted in strabismic than in anisometropic amblyopia (McKee et al., 2003).

According to the currently accepted hypothesis, amblyopia arises from the mismatch between the images to each eye; the information from one eye becomes favored while from the other eye it is suppressed (Harrad, 1996). This suppression causes reduction of visual acuity in this eye and therefore compromises binocular vision. However, it is not clear whether the visual acuity loss is the cause or the consequence of the impaired binocular function. Normal binocular vision provides a very strong visual cue for depth perception which in turn improves our ability for prehension movements : grasping and reaching (in particular the terminal reach phase) tasks (Melmoth and Grant, 2006). It has been shown that amblyopic patients indeed are impaired in planning and execution of reaching movements (Niechwiej-Szwedo et al., 2011a) and in the temporal coordination of eye-hand movements (Niechwiej-Szwedo et al., 2011b). Recently, amblyopia has been considered more as a primarily binocular disorder which motivated new approaches to treatments focusing on restoring the binocular vision.

Many studies examined the cortical network involved in the processing of depth from binocular information but only a few imaging studies have tested amblyopic patients under binocular viewing conditions. Here we review the studies focusing on the cortical processing of binocular vision and the cortical deficits in amblyopia. We highlight brain regions in which dysfunction might be related to the binocular deficits in these patients. Future work will help understand the neural plasticity mechanisms which might be involved in these brain regions in patients undergoing binocular treatments.

## BINOCULAR VISION

Animals with forward facing eyes such as primates have the ability to extract depth information from the 2D retinal images. When gazing at an object, the eyes’ horizontal separation induces projections onto the retinae which differ mainly in their horizontal positions. This difference in the retinal images is called horizontal binocular disparity. Detection of binocular disparity was demonstrated in human infants between 2 and 4 months of age, by comparing the visually evoked potentials (VEP) elicited by random-dot stereograms and classic black and white checkerboards (Petrig et al., 1981). Moreover, Yonas et al. (1987) demonstrated using a preferential looking procedure that 4-month-old infants sensitive to binocular disparity can also perceive the 3D shape from binocular depth cues. Despite a rather early start of binocular vision development (Fox et al., 1986), stereoacuity reaches adult level only between 6 and 9 years of age (Romano et al., 1975; Simons, 1981; Giaschi et al., 2013).

Non-human primates are very good animal models for investigating binocular vision in humans and therefore to understand its associated disorders. The main reason for this is that the monkey visual system is close to the human visual system in many aspects including its development and psychophysical properties of monocular (De Valois et al., 1974) and binocular visual processing (Cao and Schiller, 2002). Therefore, many of the studies reported hereafter were performed in non-human primates.

### ELECTROPHYSIOLOGICAL STUDIES

Visual information delivered from the retina of either eye remains largely independent until it reaches the cortex. Therefore the first stage of binocular disparity processing is located in the primary visual cortex (area V1; Poggio and Fischer, 1977; Cumming and Parker, 1999). Although V1 neurons encode absolute disparity they do not encode for relative disparity (Cumming and Parker, 1999). The relative disparity, which is the difference in absolute disparities, is critical for depth-structure perception as it is independent of eye position. This suggests, disparity selective neurons in V1 are not associated with stereoscopic depth perception per se (Cumming and Parker, 1997) but perhaps more involved in vergence eye movements (Masson et al., 1997). Several studies using single-cell recording techniques in monkeys have reported disparity selective neurons in extrastriate areas. Studies have described such neurons in the early visual areas V2 (Hubel and Livingstone, 1987; Poggio et al., 1988) and V3 (Felleman and Van Essen, 1987; Adams and Zeki, 2001), in the dorsal pathway in areas V3A (Anzai et al., 2011) and middle temporal (MT; Maunsell and Van Essen, 1983; DeAngelis and Newsome, 1999), in the ventral pathway in area V4 (Watanabe et al., 2002; Hegdé and Van Essen, 2005), and in the inferior temporal cortex particularly in the rostral lower bank of the superior temporal sulcus (STS; Janssen et al., 1999; Liu et al., 2004). In the parietal cortex, in particular in the lateral bank of the intraparietal sulcus (IPS), neurons encoding orientation in depth were reported in the caudal intraparietal area (CIP; Taira et al., 2000; Tsutsui et al., 2001), area LIP (lateral intraparietal; Gnadt and Mays, 1995), and area AIP (anterior intraparietal; Srivastava et al., 2009) where neurons were also recorded with selectivity to 3D depth profiles. Finally, in the frontal lobe, disparity-selective neurons were reported in the frontal eye field (FEF) area (Ferraina et al., 2000). In the ventral premotor cortex, a rather high proportion of disparity selective neurons was found (Theys et al., 2012). These neurons were found in area F5 known to house visuomotor neurons (Murata et al., 1997) and to receive projections from the parietal area AIP (Borra et al., 2008).

### BRAIN IMAGING IN HUMANS AND NON-HUMAN PRIMATES

**FIGURE 1:**
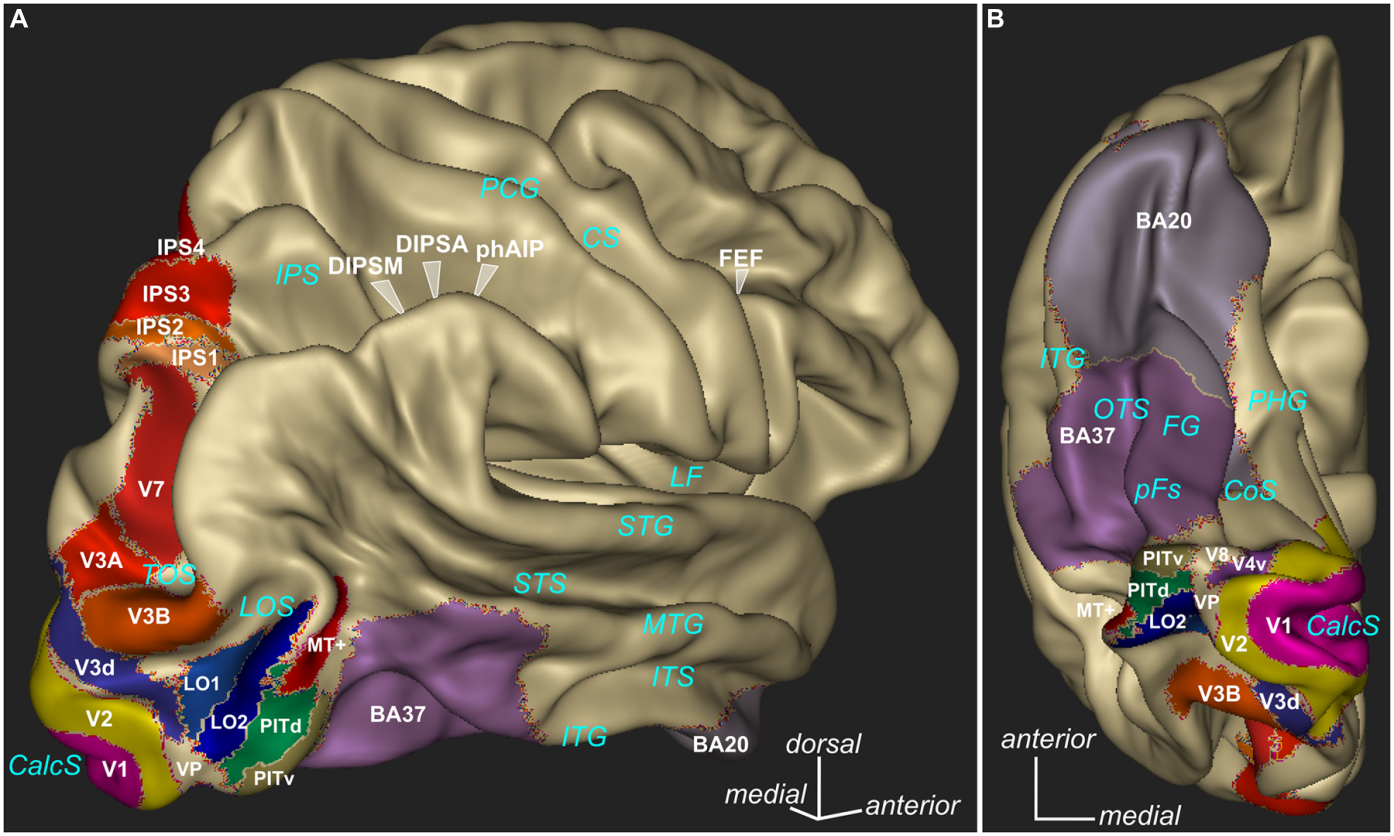
**Parcellation of different cortical regions involved in visual processing.** Some of these regions are particularly involved in binocular vision and some regions are known to show deficits in amblyopes under diverse visual stimulation. Lateral view **(A)** and ventral view **(B)** are presented. The 3D rendering (Anatomist, www.brainvisa.info) represents the cortical surface of the Conte69 human surface-based atlas (Van Essen et al., 2012). V1, V2, MT+ as defined by (Fischl et al., 2008), V3A, V3B, V4v, V7, IPS1/2/3/4 as defined by (Swisher et al., 2007), V3d, LO1, LO2, PITd, PITv, as defined by (Kolster et al., 2010), occipitotemporal area BA37, inferior temporal area BA20 available in Caret software (www.nitrc.org/projects/caret/, Van Essen et al., 2001). CalcS, calcarine sulcus; LOS, lateral occipital sulcus; TOS, transverse occipital sulcus; ITG, inferior temporal gyrus; ITS, inferior temporal sulcus; MTG, middle temporal gyrus; STS, superior temporal sulcus; STG, superior temporal gyrus; LF, lateral fissure; OTS, occipitotemporal sulcus; CoS, collateral sulcus; PHG, parahippocampal gyrus; PCG, postcentral gyrus; CS, central sulcus.

Several studies using functional magnetic resonance imaging (fMRI) in monkeys have either confirmed or predicted the above electrophysiological results. These imaging studies in non-human primates allow on the one hand a better integration of human fMRI results with the monkey single cell studies and on the other hand a possibility to assess the putative homologies between cortical areas in the two species. In the dorsal stream, Tsao et al. (2003) reported larger activations to non-zero than to zero disparity stimuli in area V3A and in the caudal intraparietal regions in both humans and monkeys. In humans, fMRI activations for 3D shape from disparity were reported in V3A and V7 (Backus et al., 2001; Georgieva et al., 2009) and fMRI adaptation to either relative or absolute disparities (Neri et al., 2004) was higher to absolute disparity in dorsal areas (V3A, MT/V5, V7) while ventral areas (hV4, V8/V4) showed a similar adaptation to both types of disparities. The role of the regions in the lateral bank of the monkey IPS in the processing of 3D shape from disparity was also investigated. Durand et al. (2007) found a difference between CIP and rostral part (anterior LIP and AIP) of the IPS in the different aspects of depth information in monkeys. In humans, several studies have clearly reported the involvement of the parietal cortex (Naganuma et al., 2005), DIPSM/DIPSA (dorsal IPS medial/anterior) and phAIP (putative human AIP) in processing of depth from disparity (Durand et al., 2009; Georgieva et al., 2009; Minini et al., 2010). In the ventral premotor cortex, imaging in monkeys (Joly et al., 2009) revealed responses to 3D surfaces in area F5a. This finding was later confirmed with electrophysiology and the report of disparity-selective neurons in this region (Theys et al., 2012). A similar frontal region was reported in humans using the same stimuli (Georgieva et al., 2009). In the ventral stream, a multi-voxel pattern analysis (MVPA) fMRI study (Preston et al., 2008) has shown that the lateral occipital area (LO) codes for the sign of depth position (near vs far) while it is invariant to the magnitude of disparity. The LO complex together with area hMT+ was shown to be particularly responding to the 3D shapes either derived from the combination of binocular disparity and perspective (Welchman et al., 2005) or defined as the correlation between fMRI signal and observers’ discrimination performance for disparity-defined shape (Chandrasekaran et al., 2007). A region in the rostral part of the lower bank of the STS in monkeys (Joly et al., 2009) and the posterior inferior temporal gyrus (ITG) in humans (Georgieva et al., 2009) were also found to be sensitive for 3D stimuli. Most of these human cortical regions that define a network for depth perception from binocular disparity (illustrated in **Figure 1**) could have impaired function in amblyopia and therefore be responsible for the impaired binocular vision detected in the patients.

## NEURAL CORRELATES OF AMBLYOPIA

The classic experiments of (Wiesel and Hubel, 1965) in kittens opened the way to examine the neural basis of visual loss in amblyopia. Both the feline and primate models of amblyopia failed to reveal significant anatomical and physiological abnormalities in the retina of the amblyopic eye (Cleland et al., 1980, 1982). Similarly in humans, many studies have described the retina as essentially normal in amblyopes (Hess and Baker, 1984; Repka et al., 2009; Al-Haddad et al., 2011; Walker et al., 2011; Birch, 2013). At the next level of visual processing, in the lateral geniculate nucleus (LGN), minor changes were reported in the morphology of the cells (Guillery, 1973; Sloper et al., 1988; Sloper and Collins, 1998). In spite of these morphological changes, electrophysiological studies in cats and monkeys, demonstrated that the LGN cells had normal spatial and temporal response properties following visual deprivation (Cleland et al., 1980, 1982; Crewther et al., 1985; Movshon et al., 1987; Sasaki et al., 1998).

### CORTEX

Studies focusing on the cortex, reported reduction in binocularly driven neurons in the primary V1, and in the number of cells driven by the amblyopic eye (Wiesel and Hubel, 1963; Kiorpes, 2006). In infant monkeys, experimentally induced blur resulted in reduced spatial resolution and selective loss of neurons tuned to high spatial frequencies (Movshon et al., 1987; Kiorpes et al., 1998). The same authors also found that the binocular cortical connections disrupted by strabismus (Löwel and Singer, 1992) can lead to the development of fixation preference for one eye (Kiorpes et al., 1998; Kiorpes and McKee, 1999). In strabismic cats, Roelfsema et al. (1994) found similar firing rates in V1 for both eyes but reduced response coordination for responses evoked through the amblyopic eye of behaviourally tested strabismic amblyopic cats. This reduced coordinated activity between neurons driven by the amblyopic eye in V1 might be the origin of the transmission failure to higher cortical areas (Fries et al., 2002; Schröder et al., 2002). Many other studies also examined the binocular interactions within V1 detecting increased binocular suppression (Smith et al., 1997; Zhang et al., 2005). This increase in suppression can be responsible for the detected reduction in binocularly driven neurons in V1, as it was shown previously that reducing the suppression by the GABA-receptor blocker bicuculline restored the binocular input to more than half of the cortical neurones (Duffy et al., 1976). Furthermore, Sengpiel et al. (2006) suggested that this increase in suppression might also be responsible for the loss of binocular summation seen in amblyopic patients. This hypothesis is further supported by the observation that binocular summation can occur if the signal strength to the fellow eye is reduced to compensate for the suppression of the amblyopic eye (Baker et al., 2007). Going further in the cortical visual processing, El-Shamayleh et al. (2010) found that in area MT fewer cells responded to the stimulation of the amblyopic eye as compared to the fellow eye in amblyopic macaques. In humans, many studies used VEPs to investigate the neural correlates of amblyopia. Most of them reported smaller amplitudes and/or abnormal latencies (Arden et al., 1974; Sokol, 1983; Kubová et al., 1996; McKerral et al., 1999) when the amblyopic eye was stimulated. A more recent study also demonstrated that the amblyopic deficit measured by VEPs correlated with the task performance (Bankó et al., 2013b). Moreover, using complex stimuli (faces), the same group found a delay of N170 relative to the early P1 component over the right hemisphere during amblyopic eye stimulation suggesting a deficit in higher visual areas involved in face perception (Bankó et al., 2013a).

### NEUROIMAGING IN HUMANS WITH AMBLYOPIA

Non-invasive neuroimaging techniques allow us to investigate the neural correlates of amblyopia in humans (see **Table 1**), and compare them to the results found in animal models. Few studies focused on the subcortical structures in amblyopic patients. Using fMRI, it was shown that the LGN had reduced responses when driven by the amblyopic eye compared with the fellow eye (Miki et al., 2003; Hess et al., 2009). However, Sherman and Guillery (2002) drew attention to the fact that only 6% of the cells in LGN convey feedforward information from the retina to the cortex, the vast majority of the cells have a modulatory function. This modulation mainly originates from layer 6 of V1 (Van Horn and Sherman, 2004) and it is more susceptible to anesthesia than the feedforward input from the retina. Hess et al. (2009) used fMRI to overcome the possible effects of anesthesia, and investigated the activity in the LGN in human amblyopes. When comparing the BOLD signal change in the LGN, they found reduced averaged and peak activity when stimulating the amblyopic eye. These findings were consistent with the results of Miki et al. (2003) when examining a single amblyopic subject. This reduced activation can result from the mild morphological changes in the LGN reported previously (Wiesel and Hubel, 1963). Another possible explanation is that the modulatory feedback connections from V1 are responsible for this reduction, modifying the input of the binocular cells in V1 already at the level of LGN. This is more consistent with the electrophysiological findings, namely that the first signs of deficit are in area V1.

**Table 1:** Summary of the fMRI studies in humans with amblyopia.

Viewing conditions	Stimuli	Abnormal regions (often reduced in AE vs FE stimulation)	Reference
Binocularly and monocularly	Sinusoidal gratings	V1 and V2	Algaze et al. (2002)
Monocularly	Sinusoidal gratings	V1 and part of V2	Goodyear et al. (2000)
Monocularly	gratings, Gabor, wedge/annulus, checkerboard	V1 and V2	Barnes et al. (2001)
Monocularly	Checkerboard patterns	V1	Choi et al. (2001)
Monocularly	Checkerboard	V1	Lee et al. (2001)
Monocularly	Face and building pictures	IOG, pFs	Lerner et al. (2003)
Monocularly	Small and large red and green line drawings of objects	V1, V2, V3 V4 and pFs for small objects	Lerner et al. (2006)
Monocularly	Checkerboard	LGN	Miki et al. (2003)
Binocularly and monocularly	Multi-colored checkerboard patterns	V1 and V2	Conner et al. (2007)
Binocularly and monocularly	Gratings, checkerboard	V3a/VP; V4/V8; lateral occipital complex (LOC)	Muckli et al. (2006)
Monocularly	Moving circle targets	MT+, Anterior IPS and FEF	Secen et al. (2011)
Monocularly	High-contrast square-wave checkerboard	Connectivity : LGN to V1 and ventral and dorsal visual stream	Li et al. (2011b)
Monocularly (binocularly for LGN localizer)	Checkerboard	LGN	Hess et al. (2009)
Monocularly	Checkerboard (low versus high contrast)	Striate and extrastriate cortex for high contrast	Hess et al. (2010a)
Monocularly	Checkerboard	Differences in boundary positions of visual areas from retinotopy	Li et al. (2007)
Monocularly	Plaid stimuli constructed from 2 gratings	Pulvinar, hMT+	Thompson et al. (2012)
Eye closed	None, resting-state	Cortical connectivity with primary visual cortex	Ding et al. (2013), Lin et al. (2012)
Monocularly	Checkerboard (effect of perceptual learning)	Increase activation in early visual areas, temporal lobes, and right cingulated gyrus	Zhai et al. (2013)
Monocularly	Checkerboard (effect of a–t DCS)	V2 and V3	Spiegel et al. (2013)
Monocularly and binocularly	Fractal noise pattern	Increased delay and reduced amplitude of HRF in calcarine region for binocular stimulation	Farivar et al. (2011)
Monocularly	Concentric ring stimulus	V3A and V5	Bonhomme et al. (2006)

Many studies therefore investigated area V1 in amblyopic patients. Early imaging studies in humans with amblyopia used positron emission tomography (Demer et al., 1988) and single photon emission computed tomography (Kabasakal et al., 1995). They reported reduced primary V1 response to the amblyopic eye compared to the fellow eye. Similarly, Choi et al. (2001) found that the amblyopic eye showed reduced activation in the calcarine sulcus using monocular presentation of black and white checkerboard patterns at different spatial and temporal frequencies. This suppression was more important for high spatial frequency in anisometropic amblyopia and for low spatial frequency in strabismic amblyopia. Lee et al. (2001) also focused on the activations in the calcarine fissure (area V1) with monocular presentation of checkerboard patterns and compared them between strabismic and anisometropic amblyopia. They found during monocular stimulation that the proportion of voxels activated by either normal or amblyopic eye was lower in the strabismic group than in the anisometropic group. The activation by higher spatial frequency stimuli is reduced in the anisometropic group, but not in the strabismic group. Goodyear et al. (2000) defined a region of interest that mainly covered area V1 and reported a reduced area (number of voxels) of activation during the stimulation of the amblyopic compared to the normal eye. In subjects with strabismic amblyopia, Barnes et al. (2001) reported reduced activation in visual areas V1 and V2. In one of the very few studies that used binocular stimulation, Algaze et al. (2002) measured in the occipital cortex the BOLD response to monocular and binocular presentation of sinusoidal gratings in amblyopic patients and compared it to the responses in controls. Monocular stimulation of the amblyopic eye induced a lower response relative to the same stimulation in the fellow eye, which is expected from the visual loss. More importantly, subjects with amblyopia showed a greater difference in activations (in terms of level and spatial extent of the activation) between binocular and monocular stimulation as compared to the control subjects, but this difference was driven by the amblyopic eye and the response to the fellow eye was close to the level of response for binocular stimulation. Similarly, Körtvélyes et al. (2012) reported that ERP responses were also statistically indistinguishable when stimulating both eyes or only the fellow eye. These results are in agreement with the increased suppression of the amblyopic eye by the fellow eye. Moreover, Farivar et al. (2011) reported delayed and reduced BOLD response in V1 for the amblyopic eye stimulation and a particularly high suppression when the fellow eye was open. More recently, Li et al. (2011b) investigated effective connectivity and reported a reduced connectivity of geniculate-striate and striate-extrastriate networks. Interestingly, the authors also found that this connectivity loss correlated with the depth of amblyopia.

Only a few studies examined the higher level visual areas in amblyopic patients. In the ventral visual stream, Muckli et al. (2006) found a reduction of responses to stimulation of the amblyopic eye in V4+/V8 and LO complex as compared to V1/V2 in both anisometropic and strabismic amblyopes. This suggests transmission failure from lower to higher visual areas.

Using more complex stimuli, Lerner et al. (2003) reported reduced activity for faces in the posterior fusiform gyrus (pFs), but normal activity for houses in the parahippocampal place area (PPA). Note that VEP measurements were also reduced for foveally presented faces (Körtvélyes et al., 2012). In a later study, the same authors (Lerner et al., 2006) mapped activations for small and large objects. They found that during amblyopic eye stimulation, not only early visual areas but also high level visual areas showed reduced activation for foveally presented small stimuli when compared to fellow eye stimulation.

Conner et al. (2007) performed retinotopic mapping under monocular and binocular viewing conditions in amblyopes and looked at the activation in the foveal representation in V1 and in extrafoveal V1 and V2. They found a particularly high suppression at the foveal representation of the amblyopic eye when the fellow eye was open.

Very little is known about the visual areas on the dorsal pathway including the motion areas MT and MST (medial superior temporal) of amblyopic subjects. In cats, the dorsal pathway seems less affected than the ventral pathway (Schröder et al., 2002). Psychophysical studies suggest that both perception of global motion and translation of vision into movement are affected in amblyopic subjects (Simmers et al., 2003, 2005), implying deficits in the dorsal visual pathway leading to the posterior parietal cortex. A study, with attentive visual tracking of moving targets (Secen et al., 2011) reported a reduced activity in area MT+ for both eyes in amblyopic patients as compared to control subjects. This reduced activation was found for passive viewing and all of the tracking conditions. Further in the dorsal pathway, in the FEF and the anterior IPS activation from the amblyopic eye was only reduced in the condition of high attentional load (tracking several targets). Beside the classic activation studies, other MR imaging studies such as resting-state functional connectivity (Lin et al., 2012; Ding et al., 2013; Wang et al., 2014) and fMRI adaptation were used to investigate the dysfunction in amblyopia. Wang et al. (2014) have reported in amblyopic patients a reduced functional connectivity between the visual areas and parietal and frontal cortices that subserve visuomotor and visual-guided actions. This indicates that amblyopia might affect a large network beyond theV1. fMRI adaptation technique which assumes that fMRI repetition suppression reflects neuronal adaptation, has been used recently (Jurcoane et al., 2009; Li et al., 2011a). In the first study, Jurcoane et al. (2009), interocular transfer of adaptation (IOTA) was measured using orientation-selective fMRI adaptation in normally sighted observers and in stereo-deficient amblyopic subjects. They found that amblyopic subjects showed consistent monoptic adaptation, but no IOTA in any striate and extrastriate cortical regions. Li et al. (2011a) reported cortical (from V1 and beyond) fMRI adaptation effects which were reduced in response to amblyopic eye stimulation.

## BINOCULAR TREATMENT IN AMBLYOPIA

For long, amblyopia was considered as a disorder of monocular vision. The treatment therefore was also based on this view. Indeed, patching or pharmacological penalisation of the normal eye resulted in improved visual acuity. However, the treatment is mainly effective in children, and has a high risk for recurrence once the patching is stopped (Bhola et al., 2006). Adults who were not treated during childhood, or whose visual acuity decreased after the patching was stopped, had very limited possibilities to regain their vision. Methods using virtual reality and 3D video games were tested as possible substitute for patching (Waddingham et al., 2006a,b; Gargantini, 2011).

A recent theory looks at amblyopia as a primarily binocular disorder and suggests that the treatments should focus on restoring the binocular vision. Baker et al. (2007) demonstrated that amblyopic patients, in contrast with the previous beliefs, can experience binocular summation. This summation can occur when the suppression of the amblyopic eye is accounted for by reducing the contrast in the fellow eye. Based on this finding and the hypothesis that amblyopia is primarily a binocular disorder, Hess and colleagues (Hess et al., 2011; To et al., 2011) proposed a new binocular treatment (for a review, see Hess et al., 2014). They first used a dichoptic coherence motion discrimination task (Hess et al., 2010b). Later they adapted the method to a popular video game (Tetris, Honolulu, HI, USA) that would capture the patients’ attention more resulting in better compliance with the training. The patients viewed the game dichoptically; part of the information (falling blocks) was presented only to the amblyopic eye with fixed contrast, whereas the other part (superficial ground plane blocks) was presented only to the fellow eye with decreased contrast. Only the less relevant deeper ground plane blocks were presented to both eyes in order to help binocular fusion. To play the game successfully, information from the two eyes had to be combined. By adjusting the contrast of stimulation to the fellow eye, patients could experience binocular summation, and play the game. Training nine adults with this dichoptic game that facilitated binocular summation, resulted in decreased suppression of the amblyopic eye, significantly greater improvements in visual acuity and stereopsis than with monocular training (Li et al., 2013). The decreased suppression was demonstrated as a decreased difference in stimulus contrast between the amblyopic and fellow eye that still allowed binocular summation.

This treatment overcomes many weaknesses of the previous treatment strategy using patching of the fellow eye, namely that it is effective in adults, well beyond the critical period of visual development, supports the binocular interaction between the eyes and increases the compliance with treatment when adapted to popular video games. Long-term follow up of the patients treated dichoptically will reveal whether this treatment would also decrease the rate of recurrence.

Another promising technique for treating amblyopia in adults can be brain stimulation. Thompson et al. (2008) have shown that repetitive transcranial magnetic stimulation (rTMS) of the V1 can temporarily improve contrast sensitivity in the V1 of adult amblyopic patients. When applied for 5 consecutive days (Clavagnier et al., 2013), rTMS was shown to have a long lasting effect (tested up to 78 days). A recent study (Spiegel et al., 2013) using brain stimulation (anodal transcranial direct current stimulation) and fMRI measurements in amblyopic patients indicated that the stimulation could equalize the response of the V1 to inputs from each eye. This latter study also suggests that fMRI could be used to understand the neural mechanisms and the brain regions involved in these therapies (e.g., Zhai et al., 2013).

## CONCLUSION

Amblyopes suffer not only from poor visual acuity but also from deficits in binocular vision. Binocular disparity, a strong visual cue for depth perception, involves many cortical regions and some of them were shown to respond abnormally in amblyopic patients. Imaging studies in amblyopia started to use binocular stimulation, however, the cortical mechanisms of the binocular impairments remain largely unknown. Binocular treatment, a very promising alternative to patching, encourages binocular summation and might involve neural plasticity in brain regions involved in binocular vision such as the parietal cortex.

## Conflict of Interest Statement

The authors declare that the research was conducted in the absence of any commercial or financial relationships that could be construed as a potential conflict of interest.
